# Assessment of Knowledge regarding Climate Change and Health among Adolescents in Yogyakarta, Indonesia

**DOI:** 10.1155/2018/9716831

**Published:** 2018-02-15

**Authors:** Sulistyawati Sulistyawati, Surahma Asti Mulasari, Tri Wahyuni Sukesi

**Affiliations:** Faculty of Public Health, Universitas Ahmad Dahlan, Jl. Prof. Dr. Soepomo, Janturan, Umbulharjo, Yogyakarta, Indonesia

## Abstract

This research was aimed at providing evidence on climate change and health knowledge among adolescents. A cross-sectional study was conducted in Yogyakarta city from June to September 2016. A structured questionnaire was used to collect data among 508 adolescents who were in the second grade of a senior high school. This study revealed that participants had a low and inconsistent understanding regarding climate change and its impact on health. They reported that they prefer to get climate change information via talking with family. In summary, adolescent knowledge regarding climate change and health needs to improve with proper content and appropriate media.

## 1. Introduction

Climate change, which is a process of changing the climate system over a long period and over a wide area due to natural processes or as a consequence of human activity [1], has become a global issue. Natural processes have a small contribution to climate change, whereas human activity is the most significant contributor. The International Panel on Climate Change (IPCC) has reported that evidence of human influence on climate system change is well defined [2]. Climate change is related to global economic developments that influence industrialization improvement, including greenhouse gases emission [3]. Greenhouse gases consist of several gases; one of them is carbon dioxide (CO_2_), which is mainly produced by fuels burning activities. The CO_2_ concentration has increased by 31% since 1750 [4], which was mainly caused by deforestation, transportation activities, and the industrial sector. Scientists noticed the changing of climate systems via some signs [4]: the global average temperature has increased by 0.6°C since 1861 and then by 0.6 ± 0.2°C throughout the 20th century. Likewise, the global sea level rose by 0.1 to 0.2 meters during the 20th century. Lastly, the snow cover and ice extent decreased by about 10%.

It is predicted that climate changes affect both the environment and the humankind, whereas human health is affected by the ecological condition. Climate changes adversely affect humans, including their health, in several ways. The rising global temperature causes problems to people who have respiratory diseases such as asthma. A research reported an increase of severe asthma incidence during a thunderstorm in the pollen season due to allergy [5]. Another case reported that high precipitation, high temperature, and wind influence people's outdoor physical activities, including young people [6]. Physical activity is one of the health determinants. Another climate change effect on health is related to vector-borne diseases. Thus, due to climate variability, economic status, vector control ability, and drug resistance [7], according to that evidence, the climate change and human health are interrelated.

As a big archipelagic state, Indonesia is one of the countries that are vulnerable to climate changes because of its geographical position and subtropical climate [8]; it also has a large population density, who rely on agriculture [9]. Sea level rise, flooding, drought, and landslides are different forms of climate change hazards that harm Indonesia. Climate change in Indonesia has been noticed from the temperature increase of about 0.3°C since 1990, and it happened in all seasons all the year [10, 11]. During precipitation, it was predicted that Indonesia will experience around 2-3% more precipitation each year due to climate change (Ratag, 2001 [10]). In the last three decades, Indonesia has experienced several disasters, 80% of which were associated with climate change [12]. This has shown that Indonesia is a very vulnerable country to climate change.

Yogyakarta is a city in Indonesia which has been facing climate change, including its impact on health. However, limited research has measured climate change and its impact in this area. The research by Retnadi Heru Jatmiko showed that the temperature in urban parts of Yogyakarta had been changing compared to the suburban area [13]; this finding indicated that the city is becoming warm. Changing of land cover was a factor that triggered the shifting of temperature in urban parts of Yogyakarta. Another climate change event that has occurred in Yogyakarta is climate extreme. Recently, Yogyakarta was hit by a cyclone which had a moderate impact on the society [14]. On that occasion, the high intensity of rain caused flooding in some areas of Yogyakarta.

Rising temperature and the occurrence of cyclones and flooding, which are varieties of climate change impacts, have affected human health in Yogyakarta. Local health authorities recorded during the last ten years the number of vector-borne diseases such as dengue fever, which has increased three times during 2001–2010 [15]. They also reported leptospirosis cases in some years [16, 17]. Both diseases may associate with high rainfall intensity in Yogyakarta as the impact of climate change.

Assessing teenagers' understanding related to climate change impact on health is a critical section for some reasons. First, it is important to have a better adaptive capacity for teenagers. As a simple example, if teenagers understand that sun exposure damages their skin, they will apply appropriate protection. Knowing the respondent's preference of climate change source information is an essential part to provide effective and efficient information. Second, a teenager or adolescent is a proper agent of change whose possible role is a climate change message carrier. Some researches have successfully engaged adolescents as a message carrier [18, 19]. Nevertheless, a limited number of studies addressed the role of adolescents in climate change, including in Indonesia. Even though research was conducted to capture the adolescent perception in a similar topic [20], it was organized in a small setting. Therefore, it is essential to generate research in a broader context.

Considering the whole background, the perception of adolescents regarding climate change impact on health is important to observe. Thus, we refer to the IPCC guideline in the adaptation process where knowledge data became one out of six components in adaptation implementation [21]. In our setting, we engaged adolescents as one community element, who may play a role in bringing climate change messages to the community as part of climate change adaptation preparation. Our research was aimed at assessing formative knowledge of adolescents about climate change impact on health and at providing recommendations of appropriate action towards increasing the young people's knowledge.

## 2. Materials and Methods

This cross-sectional study was conducted in Yogyakarta city, Indonesia. The population of our study consisted of 18,899 students in senior high school. By considering a 95% confidence level, a 0.59% margin error, and a 50% prevalence, the minimum sample size required was 376 students (Epi Info version 7). Accordingly, we divided the school into four groups: general high school, vocational high school, state high school, and private high school. Twenty schools, which were included in our sample as a representation of 83 schools in Yogyakarta city, were randomly selected. A total of 508 students who were in the second grade of senior high school were enrolled in this study.

Data were collected during June to September 2016. We contacted students that were indicated by the school to join this research in their classrooms. Prior to the study, an explanation of the study was provided, including information about the freedom to quit from the research without any punishment. For students that agreed to join this research, written informed consent was obtained from them.

The questionnaire was divided into three parts: general questions, climate change and health, and source information preference. The first part consisted of 13 questions asking about the climate change perception in the general framework. The second part concerned participants' perception about climate change and health (10 questions), such as the impact of climate change events on humans' health and their preference of information source about climate change and health. Each answer from the respondent was scored as 1 point. The third part was regarding information source preference.

Ethical approval for this study was obtained from the Ethical Review Board of Universitas Ahmad Dahlan, Yogyakarta, Indonesia.

An analysis was generated by Microsoft Excel and SPSS to express the frequency and the aggregate for each question.

## 3. Result

Respondents reckoned that climate change was not an important problem; only less than 15% of respondents marked climate change as a very important problem. Participants paid more attention to poverty and food and water scarcity. Only about 5% of the participants assumed that the level of community seriousness about climate change was a very serious problem, while in the participants' opinion, only about 7% of the respondents reckoned that climate change is a serious problem. The majority of participants said that they somewhat know the cause of climate change (79.53%), the consequence (53.94%), and the attempt to tackle climate change (59.45%). Most of them also believed that climate change is an unbreakable process due to their assumption that climate change is caused by a natural process (51.18%), not caused by human activity. Respondents relatively understood (51.18%) that CO_2_ has a high impact on climate change. Moreover, 77.36% of the respondents agreed that the industrial sector had a considerable contribution to climate change. More than 50% of the respondents disagreed with the statement that climate change evidence is not convincing. Besides, more than 60% of the respondents were aware that sea level rise and flood were kinds of climate change impact (Table 1).

Talking about climate change and health, most of the participants (>60%) understood that climate change affects human health and said that nowadays they are already facing the impact. Most of them (81.10%) agreed that air pollution influences their health. Besides that, they also agreed that floods, landslides, and heat waves, which caused mortality, are related to climate change. On the other hand, half of the respondents said that the climate change evidence is not convincing and is still debated among the society. Afterwards, more than half of the respondents (53.74%) were not aware that the increase of cardiovascular diseases correlates with climate change. Nevertheless, they recognized that climate change influences humans' mental health, infectious diseases, and the increase of food- and waterborne diseases (Table 2).

When asking about the information source regarding climate change, more than half of the respondents (53.54%) said that talking with family is their favourite source. Meanwhile, 15.16% of the respondents said that the Internet is their climate change information source (Table 3).

## 4. Discussion

Climate change is a serious problem all over the world. Accordingly, a proper attempt to minimize the impact should be addressed through mitigation and adaptation. Inadequate climate change knowledge in the society influences their capacity in mitigation and adaptation efforts. Adolescents, who are part of the society, are a group that is predicted to face climate changes in the future. On the other hand, adolescents are excellent agents of change to deliver climate change messages [18, 19]. Knowing and finding the gap in adolescents' climate change knowledge is essential to provide proper engagement with the adolescents. There is a growing opinion that knowledge is one of the key points in the community building adaptive capacity, so knowledge measurement is necessary [22].

In this study, we measured the climate change knowledge among adolescents who were in senior high school. Through a structured questionnaire, we found several knowledge weaknesses among participants. First, participants did not consider climate change as a serious problem or they had low climate change awareness. The poor understanding perhaps correlated with their opinion that climate change evidence is not convincing. This result was different from a research in the Czech Republic which identified that more than 80% of research participants (adolescents) had good climate change awareness [23]. Secondly, participants did not recognize that climate change is caused by anthropogenic factors. Thus, it can be seen from their opinion that climate change was an unbreakable process; also, they articulated that climate change is caused by a natural process. On the other hand, it is clear that humans profoundly contributed to the increases in greenhouse gases, which triggered global warming and climate change [24]. Consequently, as stated by the IPCC, the world population has to stop emitting greenhouse gases to prevent the severe climate change impact [25].

Even though participants had poor knowledge regarding the cause of climate change, they knew that climate change has a serious impact on human health. The same result was stated in a research in Bangladesh, where adolescents had a good understanding of the impact of environmental change on health [26]. However, in this research, it was identified that adolescents did not understand deeply what kind of health was affected by climate change. Thus, it can be seen from the participants' opinion that climate change only affects infectious diseases and does not impact noninfectious diseases such as cardiovascular diseases. Considering this research finding, it is necessary to include climate change science in the school curricula as mentioned in a study in Austria and Denmark, which is critical in improving climate change science education [27], including through the school system.

Participants revealed that their primary climate change information source is from family. We were surprised by the fact that, in the digital era, the majority of adolescents were still holding on to family values. This finding is consistent with a research where adolescents obtained contraception information from family, friends, and school, which are nondigital media [28]. This research was conducted in Yogyakarta which is a small city that upholds family value. Perhaps this was the reason why students, who are our respondents, said that family is the primary source of information.

The next participants' preference of information source was digital, mass, and electronic media, which were the Internet, radio, and television, scientific articles, and also newspapers and magazines. Television is a popular entertainment means in the Indonesian community due to its simplicity and cheapness. This research finding was almost similar to a research in India where about 60% of the respondents stated that the television is their climate change information source [29]. Meanwhile, using smartphones or gadgets has become a popular lifestyle also in Indonesia. Statista report said that, in 2017, smartphone users in Indonesia have increased rapidly since 2011 [30]. By 2017, it is estimated that more than 60 million people will be using smartphones in Indonesia [31], almost 24% of the total population of Indonesia. Accordingly, smartphones could be a promising tool for spreading information, including on climate change impact on health. This finding was in line with a research stating that digital media have to be taken into account as the main tool in public health promotion, not only as an additional tool [32].

Generally, participants' understanding of climate change and its impact on human health is relatively not consistent. For example, they said that climate change is caused by a natural process, but on the other hand, they agreed that the industrial sector is one of the causes of climate change. In another example, respondents said that climate change impacted human health, but they did not give sufficient answers on the impact of climate change on noninfectious diseases. Essentially, inadequate adolescent's knowledge regarding climate change affects their opinion about climate change itself, including the impact on health [27]. Improvement of adolescent knowledge should be in line with the improvement of the whole stakeholder, including decision-makers in the education sector. Our study has several limitations. First, this study was conducted in only one of several regions, so generating the result in other areas settings should be done carefully. Second, ensuring the truth of participants' answers was hard to do.

Referring to this research, we suggest that future studies develop a program that aims to improve the knowledge and awareness of the whole community through an integrated program, which will focus not only on adolescents but also on other family members and decision-makers. This program could be run in a broader setting to have a large impact on the community. Building climate change awareness in adolescents also can be done using popular media, such as television, radio, and social media, to provide climate change evidence through impressive animations. Likewise, we propose that knowledge assessment in the future will be completed with practice assessment to have a comprehensive conclusion.

## 5. Conclusion

Climate change knowledge is essential for the society, including adolescents, because it determines the social adaptive capacity. Providing proper information regarding climate change and health for young people is a valuable investment in disaster risk reduction. Developing this kind of research is useful for cities in the future since young people are part of the city's development. Overall, we are already experiencing a climate change; if we do not take a massive action, it will continue in the future. Accordingly, perhaps climate change is challenging to stop, but the impact will be worse if we do nothing.

## Figures and Tables

**Table 1:** Questions regarding climate change in the general context.

Question	*n* = 508	%
*Perception of the important problem: respondents who responded with 10, scaled between 1 and 10*		
International terrorism	74	14.57
Poverty, food and water scarcity	94	18.50
Increase of the elderly	90	17.72
Infectious disease	25	4.92
Global economic crisis	39	7.68
Global population growth	45	8.86
*Global warming and climate change*	*74*	*14.57*
Nuclear wars and armed conflicts	51	10.04
Noncommunicable disease spread	45	8.86
Modernization and globalization	81	15.94
*Perception of the level of community seriousness towards the climate change issue, scaled between 1 and 10*		
1	30	5.91
5	136	26.77
10	26	5.11
*Perception of the level of respondent seriousness towards the climate change issue, scaled between 1 and 10*		
1	30	5.91
5	74	14.57
10	36	7.09
*Various causes of climate change*		
Do not know	45	8.86
Somewhat know	404	79.53
Know a lot	58	11.42
No answer	1	0.20
*Various consequences of climate change*		
Do not know	163	32.09
Somewhat know	274	53.94
Know a lot	69	13.58
No answer	2	0.39
*Various attempts to tackle climate change*		
Do not know	124	24.41
Somewhat know	302	59.45
Know a lot	81	15.94
No answer	1	0.20
*Climate change is an unbreakable process*		
Totally disagree	22	4.33
Disagree	185	36.42
Agree	301	59.25
*CO*_*2*_ * has a low impact on climate change*		
Totally disagree	114	22.44
Disagree	260	51.18
Agree	134	22.44
*Climate change is caused by natural processes*		
Totally disagree	47	9.25
Disagree	200	39.37
Agree	260	51.18
*The industrial sector is the only cause of climate change*		
Totally disagree	28	5.51
Disagree	85	16.73
Agree	393	77.36
*Climate change evidence is not convincing*		
Totally disagree	79	15.55
Disagree	296	58.27
Agree	131	25.79
*The rise of sea level is one climate change impact*		
Totally disagree	35	6.89
Disagree	98	19.29
Agree	373	73.43
*Flood is one of the climate change impacts*		
Totally disagree	34	6.69
Disagree	146	28.74
Agree	325	63.98

**Table 2:** Questions regarding climate change and health.

Question	*n* = 508	%
*Climate change did not affect my health*		
Totally disagree	314	61.81
Disagree	65	12.80
Agree	124	24.41
No answer	5	0.98
*There is an impact of climate change on my health *		
Totally disagree	20	3.94
Disagree	50	9.84
Agree	434	85.43
No answer	4	0.79
*I am exposed to a climate change impact now*		
Totally disagree	25	4.92
Disagree	163	32.09
Agree	315	62.01
No answer	5	0.98
*Air pollution has influenced my health*		
Totally disagree	34	6.69
Disagree	58	11.42
Agree	412	81.10
No answer	4	0.79
*Climate extremes such as floods, landslides, forest fires, and heat waves caused mortality *		
Totally disagree	37	7.28
Disagree	73	14.37
Agree	393	77.36
No answer	5	0.98
*Evidence of climate change related to health is not convincing and is still controversial*		
Totally disagree	27	5.31
Disagree	183	36.02
Agree	294	57.87
No answer	4	0.79
*The increases of cardiovascular diseases are related to climate change*		
Totally disagree	39	7.68
Disagree	273	53.74
Agree	192	37.80
No answer	4	0.79
*Climate change has influenced my mental health, such as stress*		
Totally disagree	40	7.87
Disagree	175	34.45
Agree	289	56.89
No answer	4	0.79
*Climate change can increase food and waterborne diseases such as diarrhea*		
Totally disagree	39	7.68
Disagree	70	13.78
Agree	395	77.76
No answer	4	0.79
*Infectious diseases, for example, dengue fever, can possibly increase by climate change events*		
Totally disagree	34	6.69
Disagree	59	11.61
Agree	411	80.91
No answer	4	0.79

**Table 3:** Information source preference about climate change.

Media	*N* = 508	%
Talking with family	272	53.54
Internet	77	15.16
Radio and television	37	7.28
Scientific article	34	6.69
Newspaper and magazine	23	4.53
Talking with friend	21	4.13
Others: NGO, health professional, state corporation	44	8.66
